# Evaluation of the wound healing property of *Commiphora guidottii* Chiov. ex. Guid.

**DOI:** 10.1186/s12906-015-0813-2

**Published:** 2015-08-18

**Authors:** Michael Gebrehiwot, Kaleab Asres, Daniel Bisrat, Avijit Mazumder, Peter Lindemann, Franz Bucar

**Affiliations:** Department of Pharmaceutical Chemistry and Pharmacognosy, School of Pharmacy, College of Health Sciences, Addis Ababa University, P.O. Box 1176, Addis Ababa, Ethiopia; Department of Pharmaceutical Technology, Noida Institute of Engineering and Technology, 19 Knowledge Park II, Institutional Area, Greater Noida, 201306 India; Divison Pharmaceutical Biology and Pharmacology, Institute of Pharmacy, Martin-Luther-University, Halle-Wittenberg, Hoher Weg 8, D-06120 Halle, Germany; Department of Pharmacognosy, Institute of Pharmaceutical Sciences, Karl-Franzens University Graz, Universitaetsplatz 4/1, A-8010 Graz, Austria

**Keywords:** *Commiphora guidottii*, Essential oil, Resin, Wound healing, Anti-inflammatory, Antimicrobial

## Abstract

**Background:**

The traditional use of the oleo-gum-resin of *Commiphora guidottii* Chiov. ex. Guid., which is commonly called scented myrrh, for topical treatment of wound is well documented. The major objective of the present study was to investigate the essential oil and resin obtained from *C. guidottii* for their potential wound healing properties. Due to their influence on the wound healing process, the anti-inflammatory and antimicrobial activities of scented myrrh have also been investigated.

**Methods:**

Powdered oleo-gum-resin of *C. guidottii* was steam-distilled to obtain essential oil, and the resin was extracted from the marc with MeOH and filtered. The TLC fingerprint profile of the resin has been recorded by using silica gel GF_254_ as stationary phase. The essential oil components were identified and quantified by GC-MS. Ointments prepared from the essential oil (4 % v/w) and the resin (5 % w/w) were used for wound healing activity tests. Toxicity of the formulated ointments was investigated according to Draize skin irritation test. Acute anti-inflammatory effect in mice was evaluated using carrageenan induced mouse hind paw oedema model. Antimicrobial activity tests were carried out using disk diffusion and broth dilution techniques against 21 pathogenic bacterial and 4 fungal strains.

**Results:**

Ointment formulations of both the oil and resin were found to be non-irritant at the concentrations used and showed significant (*p* < 0.05-0.001) increase in wound contraction rate, shorter epithelization time and higher skin breaking strength as compared to the negative control. Overall, the antibacterial and antifungal activities of the oil and resin were comparable with the standard antibiotics ciprofloxacin and griseofulvin, respectively.

**Conclusion:**

The results confirm that scented myrrh possesses genuine wound healing activity supporting the traditional use of the plant.

## Background

The skin being a protective barrier against the outside world, any break to it must be rapidly and efficiently mended [1]. When acute wound healing does not progress in an orderly and timely manner, complications can occur; incisions can dehisce; hernias can form; anastomoses can leak; and fistulae can develop [2]. Many of the available drugs for wound management are not only expensive but also pose problems such as allergy and drug resistance [3, 4]. By and large, phytomedicines for wound healing are not only cheap and affordable, but are also safe. Thus, one-third of all traditional medicines in use are for the treatment of wounds and skin disorders, which is a very high figure as compared to only 1–3 % of modern drugs [5].

*Commiphora guidottii* Chiov. ex. Guid. (Burseraceae) is a shrub or tree growing up to 5 m tall; has greenish or brownish peeling bark and is native to Ethiopia and Somalia. It is fairly widespread in Somalia and in adjacent parts of Ogaden in Ethiopia. Its vernacular name is “hadi” (sometimes spelled “hedi” or “hethi” by collectors) in all areas where it is distributed. However, in Central Somalia it is also known as “dunkaal”. The oleo-gum-resin of *C. gudottii*, which is otherwise known as scented myrrh, is commonly called “habakhadi” in Somalia and “abeked” (Amharic) in Ethiopia [6], where it is added to cattle feed to improve milk production. The Somali people also use it as a remedy for diarrhoea and stomach complaints, to facilitate withdrawal of the placenta after childbirth and for topical treatment of wounds [6–8].

Literature review reveals that the essential oil of *C. guidottii* consists of mono- and sesquiterpens [9]. Bioassay guided fractionation of the EtOAc extract of the resin resulted in the isolation of the sesquiterpene (+)-T-cadinol, as the major bioactive component of the plant with spasmolytic and bactericidal effects [10, 11]. Although less potent than T-cadinol with respect to their smooth muscle relaxing properties in the rat aorta, additional minor compounds such as cadinane, guaiane, oplopane and eudesmane were also isolated from the more polar fraction of scented myrrh [12].

In an attempt to prove the traditional uses of scented myrrh for the treatment of wounds, Claeson *et al*. [11] investigated the antibacterial effects of its constituents against *Staphylococcus aureus*, which is one of the most common bacterial wound pathogens. Moreover, the use of the plant by traditional healers in Somalia for the treatment of general stomach complaints necessitated the experimental assessment of the latex or its components for their effects against cholera toxin-induced intestinal hyper secretion in mice [7] and also for their inhibitory effects on contractile responses in the isolated guinea-pig ileum [10]. The above studies confirmed that the constituents of scented myrrh possess genuine bactericidal and antidiarrhoeal activities. However, to date, there appears to have been no report on scientific investigation of its wound healing properties.

The main objective of the present study was therefore to evaluate the wound healing activity of the essential oil and resin obtained from scented myrrh using *in vivo* and *in vitro* experiments. Anti-inflammatory and antimicrobial actions of these plant products have also been investigated since such effects are crucial for wound healing process.

## Methods

### Plant material

The oleo-gum-resin of *C. guidottii* (gum opopponax first grade), collected from Ogaden Region, in eastern Ethiopia from September - October 2010, was purchased from the Ethiopian Natural Gum Processing and Marketing Enterprise (ENGPME), Addis Ababa, Ethiopia. According to the enterprise, first grade refers to the plant material that has passed through serious grabbling processes and almost free from foreign matters and contaminants.

### Experimental animals

Healthy Swiss albino mice of either sex (26–35 g and age of 9–11 weeks) were procured from the animal house of the Ethiopian Health and Nutrition Research Institute (EHNRI) Addis Ababa, Ethiopia. Adult Wistar albino rats (weighing 150–200 g and age of 10–11 weeks) were obtained from the animal house of the School of Pharmacy (SoP), Addis Ababa University (AAU). All animals were outbred strains but undergone several generations of inbreeding. They were not subjected to any drug or test substance investigation prior to the experiments, and were bred selectively in the ratio of 1 male to 3 females, and kept in a 12 h light/dark cycle and at room temperature, provided with commercial pelleted ration and clean water *ad libitum.* The animals were cared for feeding, watering, housing, analgesia, and any health problems at all time points and especially during experimental periods. They were acclimatized for one week before the study, and during the experiment the animals were housed individually in clean plastic cages in the SoP animal house so as to avoid biting and possible wound scratch to each other. All the animals survived and were fit to conduct the experiments. They were then assigned to either treatment or control groups by simple random allotment using lottery method. The same was done for treating animals within the same group. All the experiments were conducted in Pharmacognosy laboratory of SoP in accordance with the internationally accepted laboratory animal use, care and guideline [13] and approved by the institutional Ethics Review Board of the SoP, AAU. At the end of the experiment, animals were killed using cervical dislocation.

### Microbial strains

The samples were tested against the Gram-negative bacterial strains: *Escherichia coli* CD/99/1, *E. coli* K88, *E. coli* K99, *E. coli* 306*, E. coli* LT37, *E .coli* 872, *E. coli* ROW 7/12, *E. coli* 3:37C, *Salmonella typhi* Ty2, *Shigella boydii* D13629, *S. dysentery* 1, *S. dysentery* 8, *S. flexneri* Type 6, *S. soneii* 1, *Vibrio cholerae* 85, *V. cholerae* 293, *V. cholerae* 1313 and *V. cholerae* 1315. The Gram-positive bacterial strains used were: *Bacillus pumilus* 82, *B. subtilis* ATCC 6633 and *Staphylococcus aureus* ML 267. All the bacterial strains were procured from the Department of Technology, Jadavpur University; Central Drugs Laboratory, Kolkata and Institute of Microbial Technology, Chandigarh, India. The stains were first checked for purity on the basis of standard microbiological, cultural and biochemical tests and then used for their sensitivity towards the test samples.

Antifungal activity was performed on the fungal pathogens: *Aspergillus niger* ATCC 6275, *Candida albicans*ATCC 10231, *Penicillium funiculosum* NCTC 287 and *P. notatum* ATCC 11625. All the fungal strains were procured from the Central Drugs Laboratory, Kolkata, India.

### Extraction and fractionation

Crushed air-dried oleo-gum-resin (200 g) of *C. guidottii* was steam-distilled for 5 h to obtain the essential oil. After removal of the oil, the marc left was extracted with MeOH and filtered. The filtrate was then partitioned (3×) with equal amount of *n*-hexane using a separatory funnel, to remove the remaining oil components from the resin. The methanolic layer was then dried in a drying oven at a temperature not exceeding 40 °C and the percentage yield of the resin was calculated [14].

### Thin layer chromatography (TLC)

TLC was run on analytical Silica gel 60 F_254_ (Merck) plates using a mixture of toluene and EtOAc (98:2) as a solvent system. The distance from the base line up to the solvent front was adjusted to 8 cm and 10 µL each of the resin (5 µL/mL in ethanol) and the reference compound (thymol, 1 mg/mL in ethanol) were applied 2 cm from the bottom of the plate. After the plates were developed, the spots were detected in day light or under UV light of long wavelength (366 nm) after spraying with anisaldehyde – sulfuric acid reagent followed by heating at 110 °C for 10 min.

### Gas chromatography-mass spectrometry (GC-MS)

Capillary GC-MS was performed on a Shimadzu 2010 (Shimadzu Co., Japan) using HP5-MS (Supreme-5 ms, CS - Chromatographie Service GmbH, Langerwehe Germany 30 m × 0.32 mm) column. Conditions: carrier gas hydrogen (1.5 mL/min); sample diluted with MeOH (1:1000); splitless; injection temp. 300 °C; oven 40° for 3 min isothermal; 40-150° at 6° min^-1^; 150-320º at 10 °C min^-1^ and then hold for 3 min. RIs were calculated using co-chromatographed standard n-alkanes (C9-C29). Compounds were identified by mass spectral comparison with a commercial database (Wiley 138 K mass spectral library) and laboratory’s own database. Spectral data were compared with linear retention indices published in the literature [15–18].

### Phytochemical analysis

The resin of *C. guidottii* was screened for secondary metabolites such as flavonoids, terpenoids, polyphenols, tannins, saponins and steroidal compounds according to the method described in the literature [14, 19, 20].

### Toxicity studies

#### Acute oral toxicity

Acute oral toxicity was studied according to the method described by Gatsing *et al*. [21]. Briefly, Swiss albino mice of either sex were divided into four groups of six animals each. All animals were fasted for 15 h before administration of the test samples. Animals were given 1000, 3000 and 5000 mg/kg of the resin to the corresponding groups, by suspending the sample in 2 % Tween 80, while the negative control group was given 2 % Tween 80 only. The animals were observed for any behavioural changes (like locomotion, reaction to noise, reaction to pinch, reactivity and state of excrement) during the first 3 h after a single oral dose administration of the test samples, and were given free access to food and water. Deaths, if any, were counted within the first 48 h after administration of the extracts.

### Skin irritation test

Skin irritation test was carried out using occluded dermal irritation test [22] with slight modification. Two groups of rats (each group containing six animals) were employed for this test and two areas on the dorsal side of each rat on each side of the vertebrae (1 cm from the midline of the vertebral column) were shaved and marked before the experiment. One area was for test sample ointment and the other was left untreated to be used for comparison. Topical ointment preparation of the oil and resin was applied to the respective group. Immediately after, the area was covered by dressing gauze over which a plastic sheet (occlusive material) was placed. The covering was loosely held in contact with the skin by means of a non-irritating adhesive tape and tied across the diameter of the back of the rats with an elastic bandage. After 24 h of exposure period, the elastic bandage, the adhesive plaster, the plastic sheet and the gauze were removed by taking care not to damage the skin, and the test site was rinsed with distilled water. The animals were examined for the presence of erythema and oedema according to Draize dermal irritation scoring system [23] at intervals of 1, 24, 48 and 72 h. The degree of erythema and oedema was determined based on the scores shown in Table 1.

**Table 1:** Erythema and oedema scores used to determine the primary irritation index

Erythema	Value
No erythema	0
Very slight erythema (barely perceptible)	1
Well-defined erythema	2
Moderate to severe erythema	3
Severe erythema (beef redness)	4
Oedema formation	Value
No oedema	0
Very slight oedema (barely perceptible)	1
Slight oedema (edges of area well-defined by definite raising)	2
Moderate oedema (raised approximately 1 mm)	3
Severe oedema (extending beyond the area of exposure)	4

Primary irritation index (PII) was calculated for the test samples according to the formula shown below [22].

$$ \mathrm{P}\mathrm{I}\mathrm{I}=\frac{\sum \left(\mathrm{Erythema}\kern0.5em \mathrm{at}\kern0.5em 1,\kern0.5em 24,\kern0.5em 48\kern0.5em \mathrm{and}\kern0.5em 72\mathrm{h}\right)}{\mathrm{No}\kern0.5em \mathrm{of}\kern0.5em \mathrm{test}\kern0.5em \mathrm{sites}\kern0.5em \times \kern0.5em 4\kern0.5em \mathrm{scoring}\kern0.5em \mathrm{intervals}}+\frac{\sum \left(\mathrm{Oedema}\kern0.5em \mathrm{at}\kern0.5em 1,\kern0.5em 24,\kern0.5em 48\kern0.5em \mathrm{and}\kern0.5em 72\mathrm{h}\right)}{\mathrm{No}\kern0.5em \mathrm{of}\kern0.5em \mathrm{test}\kern0.5em \mathrm{sites}\kern0.5em \times \kern0.5em 4\kern0.5em \mathrm{scoring}\kern0.5em \mathrm{intervals}} $$

If irreversible alteration of the dermal tissue is noted in any animal, which include ulceration and clear necrosis or signs of scar tissue, the test item is classified as corrosive in which case Draize classification was not applicable.

### Ointment formulation

Simple ointment base was prepared according to the British Pharmacopoeia [24] using hard paraffin, cetostearyl alcohol, white soft paraffin and wool fat. The essential oil (4 mL) and the resin (5 g) were separately incorporated into portions of the simple ointment to make 4 % (v/w) oil and 5 % (w/w) resin ointments of uniform consistency and smooth texture.

### *In vivo* wound healing models

#### Excision wound model

Four groups of rats of either sex containing six per group (3 male and 3 female) were anaesthetized with diethyl ether. Diethyl ether was used as inhalational anesthesia using desiccators, and depth of anesthesia was monitored using vital reflexes. Circular wound of area 540 mm^2^ was inflicted and left undressed. Then, 4 % (v/w) essential oil ointment or 5 % (w/w) resin ointment for the experimental groups; 0.2 % (w/v) nitrofurazone ointment and simple ointment BP for the positive and negative control groups, respectively, were topically applied on a daily basis until the wound healed completely. Wound contraction was assessed by tracing the wound area on transparent paper (subsequently transferring it to a graph paper) every other day, and the change in wound area was calculated [25]:

$$ \%\mathrm{Wound}\kern0.5em \mathrm{contraction}=\frac{\mathrm{Healed}\kern0.5em \mathrm{area}\kern0.5em \left(\mathrm{m}{\mathrm{m}}^2\right)}{540\mathrm{m}{\mathrm{m}}^2}\times 100 $$

where, healed area = original wound area (540 mm^2^) – present wound area.

Number of days required for complete falling of scab without any residual raw wound, which indicates the period of epithelization, was noted [26].

### Incision wound model

Five groups of mice containing six animals in each group were anaesthetized and longitudinal paravertebral incisions were made 1 cm from the midline of the vertebral column [27]. A full aseptic measure was not taken and no local or systemic antimicrobials were used throughout the experiment. The wound was closed with interrupted suture, 1 cm apart with the help of black silk surgical thread and straight needle [26]. Ointments of the oil (4 %, v/w) or the resin (5 %, w/w) were applied topically on the respective test groups, while 0.2 % (w/v) nitrofurazone and simple ointment were applied for the positive and negative controls, respectively, for ten days (on a daily basis). This was done every morning at 9:00 a.m. One of the five groups was left untreated. The suture was removed on the 7^th^ day and skin breaking strength was measured by continuous water flow method on day 10 after the animals were euthanized [25, 28]. Percentage of tensile strength was calculated using the following formula [29]:

$$ \begin{array}{c}\hfill \%\mathrm{Tensile}\kern0.5em \mathrm{s}\mathrm{trength}\kern0.5em \left(\mathrm{T}\mathrm{S}\right)\kern0.5em \mathrm{of}\kern0.5em \mathrm{test}\kern0.5em \mathrm{s}\mathrm{ample}=\frac{\mathrm{TS}\kern0.5em \mathrm{of}\kern0.5em \mathrm{test}\kern0.5em \mathrm{s}\mathrm{ample}\hbox{-} \mathrm{T}\mathrm{S}\kern0.5em \mathrm{s}.\mathrm{o}}{\mathrm{TS}\kern0.5em \mathrm{s}.\mathrm{o}}\times 100\hfill \\ {}\hfill \kern0.5em \%\mathrm{Tensile}\kern0.5em \mathrm{s}\mathrm{trength}\kern0.5em \left(\mathrm{T}\mathrm{S}\right)\kern0.5em \mathrm{of}\kern0.5em \mathrm{reference}=\frac{\mathrm{TS}\kern0.5em \mathrm{of}\kern0.5em \mathrm{reference}\hbox{-} \mathrm{T}\mathrm{S}\kern0.5em \mathrm{s}.\mathrm{o}}{\mathrm{TS}\kern0.5em \mathrm{s}.\mathrm{o}}\times 100\hfill \\ {}\hfill \%\mathrm{Tensile}\kern0.5em \mathrm{s}\mathrm{trength}\kern0.5em \left(\mathrm{T}\mathrm{S}\right)\kern0.5em \mathrm{of}\kern0.5em \mathrm{s}.\mathrm{o}=\frac{\mathrm{TS}\kern0.5em \mathrm{s}.\mathrm{o}\hbox{-} \mathrm{T}\mathrm{S}\kern0.5em \mathrm{l}.\mathrm{u}}{\mathrm{TS}\kern0.5em \mathrm{l}.\mathrm{u}}\times 100\hfill \end{array} $$

where, s.o and l.u stand for simple ointment treated and left untreated groups, respectively.

### Hydroxyproline assay

Hydroxyproline (0.05 g) was dissolved in water and diluted to about 400 mL with water. Concentrated HCl (20 mL) was added and the solution was made up to 500 mL with water. The solution (100 µg/mL) was then diluted to give 5, 10 and 15 μg of hydroxyproline/mL. Triplicate solutions of each of these concentrations and blank solutions were prepared. One ml of 0.05 M CuSO_4_ was added into each test tube, followed by the addition of 1 mL of 2.5 N NaOH, and the tube contents were mixed by gentle swirling. The tubes were placed in a water bath at 40 °C for about 5 min and then 1 mL of 6 % H_2_O_2_ was added. The tubes were left in a bath for 10 min with occasional swirling and cooled with tap water, then 4 mL of 3 N H_2_SO_4_ and 2 mL of 5 % dimethylaminobenzaldehyde (DMAB) solution were added by swirling after each addition. Caps were placed on tubes, which were kept in a water bath at 70 °C for 16 min. The solutions were cooled, mixed and their absorbance measured against the blank solution at wavelength of 572 nm in 1 cm cells [30] and calibration curve was constructed.

Hydroxyproline assay was performed by inducing circular wound with approximate area of 300 mm^2^ using the procedure described in excision wound model. The wounds were treated with topical application of the oil, resin, 0.2 % nitrofurazone or simple ointment for 10 days [28]. On the 11^th^ post wounding day, the animals from each group were euthanized and the wound tissue was excised, weighed, and dried in an oven at 70 °C for 18 h, and the dry weights were noted. The tissues were treated in the same way as the standard hydroxyproline was treated, and absorbance was measured at 572 nm using a spectrophotometer (Jenway Model 6500, England). The amount of hydroxyproline in the samples was calculated by using the following equation, which was obtained from the calibration curve of the standard hydroxyproline [31]:

$$ \mathrm{A}=0.0058\times \mathrm{C}+0.0067\kern0.5em \mathrm{OR}\kern0.5em \mathrm{C}=\frac{\mathrm{A}\hbox{-} 0.0067}{0.0058} $$

where, A is absorbance and C is concentration.

### Anti-inflammatory activity

Mouse paw oedema model was used to determine the acute anti-inflammatory activity of the test substances. Following overnight fasting with free access to water, the basal volume of the right hind paw of each mouse was determined before administration of any drug using plethysmometer (Ugo Basile, 7140 Italy) [32]. Then, the animals were divided into five groups (six animals per group), such that the mean volumes of the different groups were similar. Vehicle, the standard drug (indomethacin 10 mg/kg), and the resin in three dose levels (150, 300 and 600 mg/kg) were administered orally to the respective group, 1 h before carrageenan injection. The resin and indomthacin were separately suspended in 2 % Tween 80. Paw swelling was induced by sub-plantar injection of 0.05 mL of 1 % carrageenan in 0.9 % saline (w/v) into the right hind paw. Thereafter, paw oedema was measured at 1, 2, 3 and 4 h after carrageenan injection using plethysmometer [33]. Percent inhibition of oedema was calculated using the formula:

$$ \%\mathrm{Oedema}\kern0.5em \mathrm{inhibition}=1\hbox{-} \left(\frac{\mathrm{Oedema}\kern0.5em \mathrm{of}\kern0.5em \mathrm{test}\kern0.5em \mathrm{sample}}{\mathrm{Oedema}\kern0.5em \mathrm{of}\kern0.5em \mathrm{control}}\right)\times 100 $$

### Antimicrobial activity

#### Antibacterial test

Zones of inhibition produced by the samples were determined by disc diffusion technique, and compared with those produced by ciprofloxacin [34]. The resin and the oil were separately dissolved in dimethylsulfoxide (DMSO), (DMSO was found to be inactive at the concentration used). Serial nutrient agar plates were prepared and incubated at 37 °C for 24 h to check for any contamination. Sterile filter paper discs of 6 mm diameter were soaked in stock solution (200 µg/mL) of the samples (each 6 mm disc was shown to absorb 25 µL of the sample solution of reference drug stock solution in order to be saturated) and placed in the appropriate position on the surface of the plate flooded with 24 h old culture grown on nutrient broth, marked as quadrant on the back of the Petri dishes. They were then incubated at 37 oC for 24 h and the diameters of zone of inhibition were measured in mm. A similar procedure was adopted for pure ciprofloxacin (dissolved in water at a concentration of 200 µg/mL) and the zone of inhibition was compared accordingly. The minimum inhibitory concentrations (MICs) of the test samples were also determined by the checkerboard technique using nutrient agar media [35].

### Antifungal test

Antifungal activity was first evaluated by estimation of the MIC of the test substances against the fungal pathogens, followed by the disc diffusion method, using griseofulvin (at a concentration of 2000 µg/mL) as a reference standard on Sabourds Dextrose Agar (SDA) medium according to CLSI [36]. The concentrations selected for MIC determination ranges from 50 to 2000 µg/mL and the zones of inhibition were studied at the highest MIC value as described by Mitchell and Carter [34].

### Statistical analysis

Raw data obtained from most of the experiments were expressed as mean ± SEM (standard error of the mean). Results were statistically analyzed using Microsoft excel 2010 and one-way ANOVA followed by Post Hoc Tukey and Dunnett t-tests using SPSS version 20 software. Results were considered as significant at *P* < 0.05.

## Results

### Extraction and chromatography

**Fig. 1:**
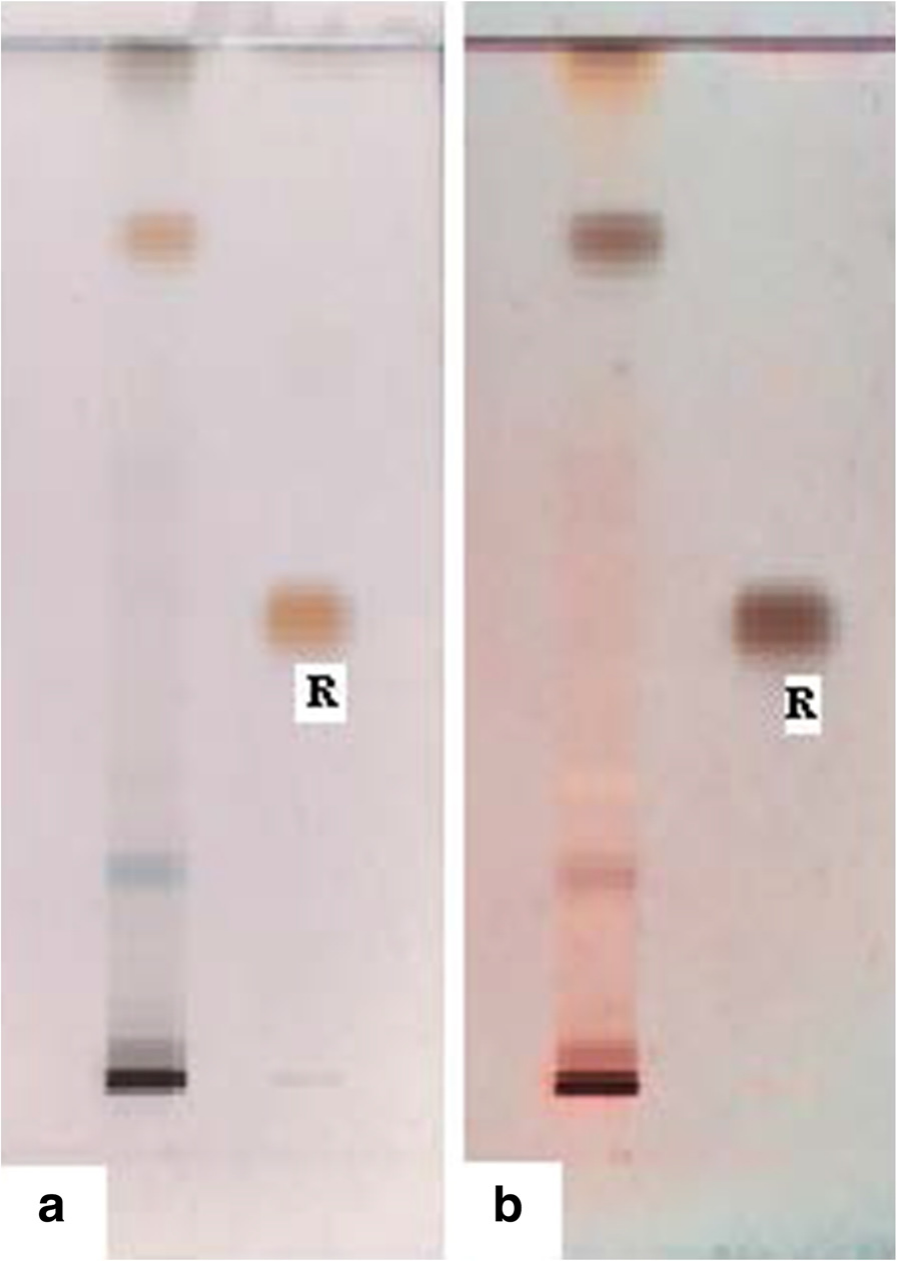
TLC fingerprint profile of the resin of *Commiphora guidottii*(solvent system: toluene/EtOAc (98:2); spray reagent anisaldehyde – sulfuric acid reagent); R: Reference (thymol): (**a**) under visible light after spray and (**b**) under UV light of 366 nm after spray

Steam distillation of the oleo-gum-resin of *C. guidottii* followed by maceration with methanol yielded a pale yellow oil (2.8 % v/w), and a reddish brown resin (39 % w/w), respectively. Preliminary phytochemical screening indicated the presence of terpenoids, steroids and phenolic compounds in the resin. These results were consistent with previous report of Hanuš *et al.* [9]. TLC fingerprint profile of the resin is depicted in Fig. 1. Chemical composition of the essential oil of *C. guidottii* was studied using GC-MS and the results are summarized in Table 2.

**Table 2:** Composition of the essential oil of *Commiphora gudottii* oleo-gum-resin

No.	Compound^a^	RI^b^	Percentage
1	*cis*-β-Ocimene	1038	0.15
2	*trans*-β-Ocimene	1044	6.71
3	Rosefuran	1100	0.26
4	α-Pinene oxide	1101	0.47
5	6-Methyl-3,5-heptadiene-2-one	1104	0.29
6	Limona ketone	1125	1.19
7	*cis*-Myroxide	1141	0.67
8	Limonen-10-ol	1288	0.50
9	1-Decanol	1328	0.53
10	α-Copaene	1374	0.18
11	β -Bourbonene	1387	0.61
12	β-Elemene	1389	3.87
13	α-Santalene	1416	19.54
14	*trans*-α-Bergamotene	1432	9.30
15	α-Humulene	1452	0.45
16	*cis*-β-Farnesene	1454	0.62
17	β-Santalene	1462	0.71
18	Germacrene D	1484	0.75
19	Sesqiterpene^c^	1494	2.29
20	α-Selinene	1498	0.97
21	Curzerene	1499	11.38
22	α-Bisabolene	1506	2.33
23	Sesqiterpene^c^	1570	1.23
24	Sesqiterpene^c^	1611	4.77
25	108, 214, 274^c^	1626	0.98
26	Furanoeudesma-1,3-diene	1654	18.63
26	Isofuranodiene	1658	6.76
27	Furanodiene	1690	1.28
28	8-α-Ethoxyisofuranodiene	1705	0.96
29	8-α-Methoxyfurandione	1848	0.60
	Total (%)		98.98

^a^Compounds listed in order of elution; ^b^RI (retention index) measured relative to n-alkanes (C9–C28) on HP5-MS column under conditions listed in the Experimental section; ^c^Compound not identified (figures represent prominent peaks in the mass spectrum)

The relative percentage of the 29 compounds (about 98.98 % of the oil) identified in the oil is also shown.

### Toxicity studies

In acute toxicity study, the resin killed more than 50 % of the animals at a dose of 5000 mg/kg, while no mortality was observed at a dose of 3000 mg/kg, except for some decrease in locomotor activity. This indicates that the dose causing 50 % death of the animals (LD_50_) is near 3000 mg/kg, indicating good safety margin. This result complements the previous report which confirmed that the non-polar extracts of the resins of *C. guidottii* are non-toxic to experimental animals up to the tested dose of 1.2 g/kg [37].

Both the formulated oil and resin of *C. guidottii* were found to cause no erythema or oedema. Therefore, their PII values were zero implying the non irritant nature of the test samples as per the dermal irritation scoring system of Draize [23] and Environmental Protection Authority [36].

### Wound healing activity

#### Wound contraction and period of epithelization

In the present study, diethyl ether was used as an anaesthetic agent because of its availability, rapid recovery, return from anaesthetic hypothermia, lower incidence of death, quick elimination from the lungs and relative technical ease of administration. Moreover, diethyl ether has a smaller impact on the receptors located in the membrane of erythrocytes maintaining a stable immune function in mice, which is a big advantage to our work since immunosuppression could have seriously affected our results.

**Fig. 2:**
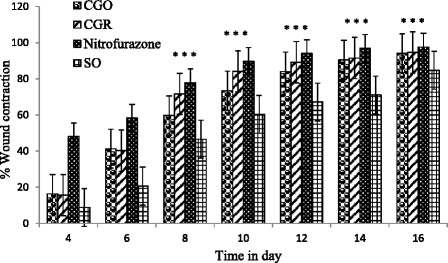
Percentage contraction of the wound of animals treated with ointments containing 4 % (v/w) essential oil or 5 % (w/w) resin of *Commiphora guidottii* (^*^Significant (*p* < 0.05); CGO: *C. guidottii* essential oil; CGR: *C. guidottii* resin; SO: Simple ointment)

As shown in Fig. 2, wound contraction was promoted till day 16 in both test substance and standard ointment treated groups. The test samples facilitated wound contraction significantly (*p* < 0.05) from day 8 to 16 as compared to the negative control, and the difference was insignificant with nitrofurazone. Time for complete epithelization was significantly (*p* < 0.05) shorter in test sample and nitrofurazone treated groups as compared to the negative control (Table 3). However, there was no significant difference between nitrofurazone and resin, between nitrofurazone and the oil, and between resin and the oil.

**Table 3:** Effect of topical application of ointments containing 4 % (v/w) essential oil or 5 % (w/w) resin of *Commiphora guidottii*on wound epithelization period of excision wound model in comparison with nitrofurazone and simple ointments

Group	Period of epithelization (day)
Essential oil	22.42 ± 0.61^*^
Resin	23.50 ± 0.72^*^
Nitrofurazone	20.67 ± 0.33^**^
Simple ointment	25.67 ± 0.42

^*^The mean value is significant (*p* < 0.01) and (^**^ *p* < 0.001) when compared with simple ointment treated group; *N* = 6; Results are expressed as mean ± SEM

### Tensile strength

The wound breaking strength of all animals treated with test samples showed significant (*p* < 0.05) difference when compared to the animals that are left untreated. Particularly, the resin showed significant (*p* < 0.05) difference in tensile strength when compared to the simple ointment treated groups. Animals treated with simple ointment increased the tensile strength by about 19.73 %, which failed to reach statistical significance, when compared to the untreated controls (Table 4).

**Table 4:** Effect of topical application of ointments containing 4 % (v/w) essential oil or 5 % (w/w) resin of *Commiphora guidottii*on wound tensile strength of incision wound model in comparison with nitrofurazone and simple ointment

Group	Tensile strength (g)	% Tensile strength
Essential oil	234.67 ± 23.40^*^	29.42
Resin	293.83 ± 53.50^*a^	78.17
Left untreated	123.00 ± 0.41	-
Nitrofurazone	319.83 ± 32.26^*a^	117.08
Simple ointment	147.33 ± 9.0	19.73

^*^The mean difference is significant (*p* < 0.05) when compared to the left untreated group; ^a^when compared to the simple ointment treated group; *N* = 6; Results are expressed as mean ± SEM

### Hydroxyproline assay

Table 5 shows the hydroxyproline content in the granulation tissues of the test animals on day 11. Hydroxyproline levels of oil and resin ointment treated groups were significantly (*p* < 0.05) increased as compared to the simple ointment treated group.

**Table 5:** Effect of topical application of ointments containing 4 % (v/w) essential oil or 5 % (w/w) resin of *Commiphora guidottii* on dry weight and hydroxyproline content of the granulation tissue in comparison with nitrofurazone and simple ointment

Group	Dry weight (g)	Hydroxyproline (μg/g of tissue)
Essential oil	41.56 ± 5.71	77.50 ± 2.46^*^
Resin	63.12 ± 14.18	63.10 ± 2.61^*^
Nitrofurazone	44.60 ± 5.01	134.50 ± 14.82^*^
Simple ointment	30.38 ± 2.18	34.00 ± 1.13

^*^The mean difference is significant (*p* < 0.05) when compared to the control group; *N* = 6; Results are expressed as mean ± SEM

### Anti-inflammatory activity

After administration of carrageenan, resin and indomethacin treated animals showed significant (*p <* 0.001) reduction of oedema as compared to the negative control (Table 6). However, there was no significant difference with the standard drug indomethacin in any course of the study.

**Table 6:** Anti-inflammatory activity of the resin of *Commiphora guidottii* on carrageenan-induced mice paw oedema

Test substance	Dose (mg/kg)	Percent oedema inhibition			
		1 h	2 h	3 h	4 h
	150	9.84 ± 0.02	32.86 ± 0.04^*^	36.23 ± 0.05^*^	53.85 ± 0.03^*^
Resin	300	21.16 ± 0.007^*^	44.98 ± 0.005^*^	54.83 ± 0.005^*^	60.05 ± 0.009^*^
	600	17.20 ± 0.006^*^	41.10 ± 0.007^*^	52.90 ± 0.008^*^	65.44 ± 0.008^*^
Indomthacin	10	27.78 ± 0.008^*^	55.00 ± 0.006^*^	68.84 ± 0.008^*^	86.52 ± 0.006^*^
Vehicle	-	19.05 ± 0.002	30.14 ± 0.006	26.06 ± 0.005	25.00 ± 0.006

^*^The mean value is significant (*p* < 0.001) when compared with the negative control group; *N* = 6; Results are expressed as mean ± SEM

### Antimicrobial activity

Zone of inhibition and MIC values of the resin and oil against different bacterial strains are shown in Table 7. The Gram‐negative bacteria including all strains of *E. coli* and *V. cholerae*, as well as *S. typhi* Ty2 were found to be the most inhibited bacterial pathogens by both the oil and resin of *C. guidottii*. The remaining Gram‐negative bacterial strains namely, all strains of *Shigella* and the Gram‐positive bacterial strains: *B. pumilus*, and *B. subtilis* were inhibited moderately. The oil and resin also exhibited good antibacterial effect against *S. aureus* with MIC values of 25 and 50 µg/mL, respectively. In general, the test substances showed low MIC (≤100 µg/mL) values against most of the tested pathogenic bacterial strains indicating their broad spectrum of action.

**Table 7:** Minimum inhibitory concentrations (MICs) and zones of inhibition (at a concentration of 200 μg/mL around a 6 mm disc diameter) of the essential oil and resin of *Commiphora guidottii* as compared with ciprofloxacin

Bacterial strain	MIC (μg/mL)^*^		Zone of inhibition (mm)		
	Oil	Resin	Oil^**^	Resin^**^	Ciprofloxacin.
*Bacillus pumilus* 82	50	200	10.5 (55.3)	11.0 (57.9)	19.0
*B. subtilis* ATCC6633	100	200	10.0 (55.6)	11.0 (61.1)	18.0
*Escherichia coli* 3:37C	25	25	14.5 (93.5)	14.5 (93.5)	15.5
*E. coli* 306	25	25	15.5 (93.9)	14.5 (87.9)	16.5
*E. coli* 872	25	25	15.5 (96.9)	14.0 (87.5)	16.0
*E. coli* CD/99/1	25	25	16.0 (94.1)	15.0 (88.2)	17.0
*E. coli* K88	25	25	16.5 (97.1)	14.5 (85.3)	17.0
*E. coli* K99	25	25	16.5 (97.1)	14.0 (85.3)	16.0
*E. coli* LT37	25	25	15.0 (93.8)	14.0 (87.5)	16.0
*E. coli* ROW 7/12	25	25	16.0 (97.0)	14.0 (84.8)	16.5
*Salmonella typhi* Ty2	10	25	15.5 (96.9)	16.0 (100.0)	16.0
*Shigella boydii* D13629	50	100	16.5 (82.5)	14.5 (72.5)	20.0
*S. dysentery* 1	50	100	16.5 (80.5)	14.5 (70.7)	20.5
*S. dysentery* 8	50	100	17.0 (81.0)	14.5 (69.0)	21.0
*S. flexneri* Type 6	50	100	16.5 (80.5)	15.0 (73.2)	20.5
*S. soneii* 1	50	100	16.0 (82.1)	14.0 (71.8)	19.5
*Staphylococcus aureus* ML 267	25	50	11.0 (61.1)	15.0 (83.3)	18.0
*Vibrio cholerae* 85	10	25	16.5 (91.7)	16.0 (88.9)	18.0
*V. cholerae* 293	10	25	16.5 (94.3)	16.0 (91.4)	17.5
*V. cholerae* 1313	10	25	16.0 (94.1)	15.5 (91.2)	17.0
*V. cholerae* 1315	10	25	17.0 (94.4)	16.5 (91.7)	18.0

^*^MICs are expressed as an average from three independent experiments, each performed in triplicate. ^**^Values in parenthesis indicate % activity of the test samples compared with that of ciprofloxacin

Similarly, the antifungal activity was studied and their MIC values determined (Table 8). Lowest MIC value was recorded for the oil (400 µg/mL) on *C. albicans* ATCC 10231. Overall, both the oil and the resin were 68.8 – 88.9 % as active as the standard antifungal agent griseofulvin.

**Table 8:** Minimum inhibitory concentrations (MICs) and zones of inhibition (at a concentration of 2000 μg/mL around a 6 mm disc diameter) of the essential oil and resin obtained from *Commiphora guidottii* against four fungal strains

Fungal strain	MIC (μg/mL)^*^		Zone of inhibition (mm)		
	Oil	Resin	Oil^**^	Resin^**^	Griseofulvin
*Aspergillus niger* ATCC 6275	1000	1500	12.5 (83.3)	11.0 (73.3)	15
*Candida albicans* ATCC 10231	400	1000	14.0 (87.5)	11.0 (68.8)	16
*Penicillium funiculosum* NCTC 287	1000	2000	12.0 (85.7)	10.0 (71.4)	14
*P. notatum* ATCC 11625	1000	2000	12.0 (88.9)	10.5 (77.8)	13.5

^*^MIC are expressed as an average from three independent experiments, each performed in triplicate. ^**^Values in parenthesis indicate % activity of the test samples as compared with that of the standard griseofulvin

## Discussion

Characteristic TLC fingerprinting of an extract not only helps in the identification and quality control of a particular plant species but also provides vital information for isolation and identification of chemical marker(s). Thus, TLC fingerprint of the resin of *C. guidottii* given in this work may serve as a reference for proper authentication, standardization and quality control of the plant drug. GC-MS analysis confirmed that the major constituents of the essential oil of *C. guidottii* is α-santalene, although furanoeudesma-1,3-diene and curzerene also occurred in large amounts (Table 2). Previous report by Craveiro *et al*. [38] indicates that α-santalene along with α-bisabolene and furanodiene are the major components of the oil. However, in the present study the later two compounds were detected in lesser amounts.

The wound contraction ability of the oil and resin of scented myrrh observed in this study indicates that the plant possesses a definite pro-healing action, since 88 % of the healing of wound occur due to contraction, and the other 22 % occur due to scar formation [39]. One reason for the wound contraction ability of these plant products could be through enhancing the proliferation of epithelial cells [40]. On the other hand, since incision wound treated with the test samples showed greater tensile strength as compared to the negative control, it might be inferred that the test samples not only increased collagen synthesis per cell, but also aided in cross-linking of the proteins [3, 26, 41]. The common method of collagen determination is based on the quantification of hydroxyproline which accounts for approximately 10 % of the collagen molecule [42, 43]. It is known that collagen accumulation is the sum of synthesis and destruction occurring simultaneously during wound healing process [44]. Therefore, the increased amount of hydroxyproline in the study groups indicates increased collagen content whereas, collagen content in the negative control group was relatively smaller, which might be due to a prolonged inflammatory phase, where the degradation of collagen is greater than its synthesis.

Inflammation induced by carrageenan develops immediately following injections, and produces three distinct phases. The first phase (0–1.5 h) is mediated by histamine and serotonin. The second phase (1.5–2.5 h) is mediated by bradykinin, while prostaglandins are implied in the third phase (2.5–5 h). The third phase of oedema is sensitive to most clinically effective anti-inflammatory drugs, which have been frequently used to assess the antioedematous effect of natural products [33, 45]. Accordingly, the resin of *C. guidottii* showed higher percentage inhibition of oedema formation (>50 %) 3 h after injection of carrageenan, which may lead to a conclusion that its anti-inflammatory activity is through inhibition of prostaglandin synthesis.

As shown in Table 7, both the resin and essential oil of *C. guidottii* displayed broad spectrum antibacterial activity. However, their activity against the Gram‐negative bacteria tested was much higher than their effect against the Gram-positive ones. Generally, Gram‐negative bacteria are more resistant to antimicrobial agents since they are covered with a phospholipid membrane, carrying the structural lipopolysaccharide component that makes their cell wall impermeable to antimicrobial substances [46]. In the present study, however, higher activity was noted against the Gram-negative bacteria. This might be due to the ability of scented myrrh constituents to affect the overall impermeability and integrity of the bacterial cell wall. In general, the activity obtained may be regarded as highly significant, since plant extracts having MIC values ≤ 8000 µg/mL are considered to be effective antibacterial agents [47]. It is particularly interesting to note that both the oil and resin showed good activity against *S. aureus* (MIC = 25 and 50 µg/mL, respectively), which is one of the most common bacterial wound pathogens. The antifungal activity of the test samples is also very important in wound healing since fungal superinfection may be present in some cases. More often than not, topical agents providing both antibacterial and antifungal activity are highly desired for wound healing [48]. Postoperative wounds are known to be complicated by infection ultimately delaying angiogenesis [49, 50]. Microbes can also cause poor quality granulation tissue formation, reduced tensile strength of connective tissue, impaired epithelization and odor [51].

When we look at the phytoconstituents, the wound healing activity of *C. guidottii* is likely to be attributed to its high content of terpenoids, since terpenoids are known to promote the wound healing process, mainly due to their astringent and antimicrobial properties, which could be responsible for wound contraction and increased rate of epithelization [20]. Sesquiterpene lactones are known to possess antioxidant activity, which may contribute to the wound healing process [49] and triterpenes are also responsible for promotion of rapid wound healing [50].

According to Biswas *et al*. [51], an ideal drug for wound should fulfill the criteria such as rapid contraction of wound leading to quick healing, reduction of wound epithelization time and appreciable gain of tensile strength. It has been reported that a plant based remedy should affect at least two different processes of wound healing before it can be said to have some scientific support for its traditional use as a wound healing agent [52]. In this regard, it is fair to say that the formulated ointments of *C. guidotti* oil and resin fulfill these criteria as they increased wound contraction and tensile strength besides reducing epithelization time. These results together with the genuine anti-inflammatory and antimicrobial effects observed make these plant products candidates for the development of efficient wound healing drugs, although the reproducibility of some of the experiments could to some degree be affected by the subjectivity involved in some of the observations like skin irritation test and determination of period of epithelization.

## Conclusion

From the foregoing, it can be concluded that the wound-healing potential of scented myrrh may be attributed in full or in part to the presence of terpenoids, which may act individually or possess additive effect that accelerates the healing process. Furthermore, the broad spectrum antimicrobial activities of the oil and the resin as well as the anti-inflammatory effect of the resin may contribute to the overall wound healing capacity of the plant. In view of close similarities between rodents and human biology, and the relative safety of the resin in mice coupled with the mere absence of irritation upon topical application of the formulated ointments on the skin, scented myrrh could be a good candidate for the preparation of natural therapeutic agents for wound management, supporting the its traditional use as a remedy for wounds. However, further studies on histology of tissue obtained from treated group and the expression of growth factors are highly warranted to show the changes in the tissue architectures at different stages of wound healing and to understand the mechanism of action of the resin and the oil in wound healing process, respectively.
